# Modeling and measurement of curing properties of photocurable polymer containing magnetic particles and microcapsules

**DOI:** 10.1038/micronano.2017.35

**Published:** 2017-08-21

**Authors:** Masato Yasui, Koji Ikuta

**Affiliations:** 1 Laboratory for Cell Signaling Dynamics, RIKEN Quantitative Biology Center, 6-2-3 Furuedai, Suita, Osaka 565-0874, Japan; 2 Graduate School of Information Science and Technology, The University of Tokyo, 7-3-1 Hongo, Bunkyo-ku, Tokyo 113-8685, Japan

**Keywords:** Magnetic particles, Microcapsules, Microstereolithography, Photocurable model, Photocurable polymer, Resin

## Abstract

In microstereolithography, three-dimensional microstructures are created by scanning an ultraviolet laser on a photocurable resin and stacking several such layers to form the desired structure. By mixing different types of particles in the resin, the formed microstructures exhibit various physical properties. For example, the magnetism and density of the microstructure can be controlled by adding magnetic particles and microcapsules to the resin. This method has been used to fabricate magnetic micromachines. Although such functional resins are useful, the incorporated magnetic particles and microcapsules can affect the fabrication resolution, making it difficult to fabricate microstructures with high precision. Thus, it is necessary to understand the effects of such microparticles and microcapsules on the fabrication process. In this study, we propose a simple model of curing resins containing magnetic particles and microcapsules to explain the effects of the magnetic particles and microcapsules. The proposed model can explain the observed curing characteristics of a resin that contains particles for all concentrations as well as for different types of magnetic particles and microcapsules. Finally, using the proposed model, we discuss how to improve the characteristics of resins containing microparticles to realize the high-resolution fabrication of three-dimensional microstructures with desirable material properties.

## Introduction

Three-dimensional (3D) printing enables the fabrication of complex 3D structures and is widely used in many fields. For example, 3D printing is being employed for tissue engineering for medical applications^1^. Cells can be positioned using 3D printing, with the aim of constructing living tissue or organs. In addition, 3D printing is being used for the reconstruction of organs based on magnetic resonance imaging, which helps doctors clearly understand the 3D structure of organs and bones^2^.

Three-dimensional printing involves several fabrication methods, such as stereolithography, selective laser sintering, and fused deposition modeling^3^. In stereolithography, a lens-focused ultraviolet (UV) laser beam is scanned on the surface of a photocurable resin to form an arbitrary two-dimensional plane. By stacking such two-dimensional planes, a 3D structure can be realized. In selective laser sintering, a powder instead of a photocurable polymer is used as the starting material. The powder is melted by the laser and allowed to solidify to form the desired structure. Unlike stereolithography, structures can be modeled using metals instead of polymers. In fused deposition molding, a molten polymer is discharged from a nozzle and allowed to solidify to form a structure. By scanning the nozzle three-dimensionally, it is possible to form any 3D structure. However, the resolution of this method is lower than that of the laser-based modeling methods. Therefore, this method is suitable only for modeling relatively large structures. Out of these fabrication processes, stereolithography has the highest resolution. The diameter and depth resolution can be as high as 12 and 7 μm, respectively^4^. High-resolution stereolithography is called microstereolithography.

By using functional photocurable polymers, it is possible to modify the physical properties of the fabricated 3D microstructures, and several types of micromachines have been developed using this technique. For example, an electrically conductive photocurable polymer has been developed, and a microactuator based on electrostatic interactions was created using this polymer^5^. In this microactuator, the grip of two electrically conductive photocured polymer beams is controlled by applying a voltage between the beams. Because of the electrostatic force between the beams, the opening and closing functions can be controlled based on the applied voltage. Furthermore, a conductive metal structure was created by adding a metallic powder to a photocurable resin and then evaporating the cured resin^6^. In addition, 3D structures including ceramics have been created by subjecting a resin containing ceramic particles to microstereolithography^7^.

Our group has developed a magnetically photocurable polymer that was used to fabricate a magnetic micromachine^4^. Because this magnetically photocurable polymer contains a viscosity-increasing agent to prevent the aggregation of the magnetic particles present in the polymer, structures with a high concentration of magnetic particles can be created. This magnetically photocurable polymer was used to produce a microscrew pump and a screw-type magnetic micromachine^8^. Furthermore, we have developed a magnetically photocurable polymer, and its density can be adjusted to enable the 3D control of magnetic micromachines^9^. This polymer contains magnetic particles and hollow microcapsules. Because the density of the hollow microcapsules is low, it is possible to reduce the density of the resin. Using this polymer, it is possible to produce 3D microstructures that exhibit a neutral buoyancy and can be controlled in a 3D manner in water.

Although these functional polymers are useful for fabricating micromachines, magnetically controllable resins and density-controllable resins present problems during 3D fabrication. The presence of magnetic particles and microcapsules changes the photocuring properties of the resin^4,9^. To overcome this problem, it is necessary to elucidate the effects of the magnetic particles and hollow microcapsules on the curing characteristics of resins. Experimental and theoretical studies have already been conducted on photocurable resins containing ceramic particles^10^. In the model employed in these studies, Mie theory was used to simulate light scattering, whereas the intensity of the light scattered and the chemical reactions involving the resin were simulated using the Monte Carlo ray-tracing technique. These analytical methods are effective in obtaining accurate results for common microparticle types. However, the relationship between the photocuring properties and materials is complicated, and it is not easy for material developers to determine which characteristics of the microparticles should be improved. Therefore, a simple model that conveniently indicates the physical properties that should be changed and the amount by which they should be changed is beneficial.

In this study, we built a simple model to investigate the relationship between the photocurable property of a resin containing microparticles and the physical properties of the microparticles with the objective of understanding and thereby improving the functional and photocuring properties of functional polymers. We verified the accuracy of our model by measuring the photocurable properties of a resin that contains magnetic particles and microcapsules. Finally, we demonstrated the key properties for fabricating a functional photocurable polymer that has high photocuring and functional properties.

## Materials and methods

### Materials for resin containing magnetic particles and microcapsules

We used SCR770 (D-MEC, Ltd., Tokyo, Japan) containing a viscosity-increasing agent (D-MEC) as the test resin in this study. Three types of magnetic particles, namely, ferrite (FA-700), a rare metal (SeFeN), and magnetite particles, were added to the resin. These particles were purchased from Toda Kogyo Corp (Hiroshima city, Japan). The microcapsules (FN-80SDE) used were purchased from Matsumoto Yushi-Seiyaku Co., Ltd, Osaka, Japan. The densities and diameters of the magnetic particles, microcapsules, and resin used are listed in Table 1.

### Measurement of curing depth and width

**Figure 1:**
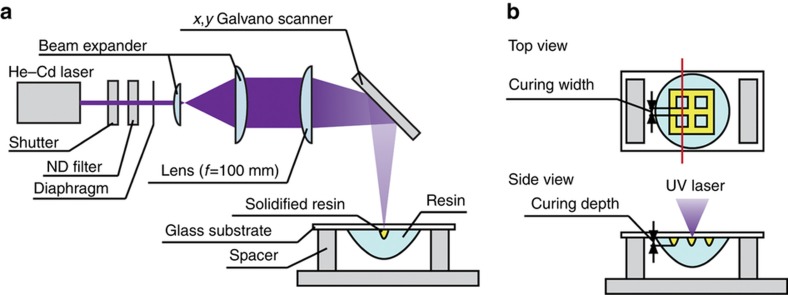
Measuring the curing depth and width. (**a**) Optical system used for measuring the curing characteristics. The wavelength of the He–Cd laser was 325 nm. The focal length of the lens was 100 mm. (**b**) Schematic of the experimental setup used for measuring the curing depth and width. The mesh structure was fabricated by scanning the ultraviolet (UV) laser. The upper figure is the top view of the scanned resin sample. The curing width is the width of the cured resin sample along the red line in the upper figure. The lower image shows the cross-section along the red line in the upper figure. The curing depth is the depth to which the resin was cured (see lower figure).

To measure the curing characteristics of the resin samples containing the magnetic particles, we used the UV laser-scanning system shown in Figure 1a. In this system, to realize a high numerical aperture, the beam diameter is increased by the beam expander before the beam passes through the condenser lens. The expanded laser beam is focused using a condenser lens with a focal length of 100 mm. The focused laser beam is scanned using an *x*–*y* galvano-scanner. Furthermore, a mechanical shutter is used to switch the UV laser on and off. The light intensity is controlled by the neutral-density filter. The power of the laser is measured in front of the beam expander.

The protocol used for measuring the curing depth and width of the resin was as follows. The mixed resin was placed on a glass substrate, and the substrate was placed on the UV laser-scanning system. The focused UV laser was scanned over the glass substrate to fabricate a meshed structure, as shown in Figure 1b. After completion of the scanning process, the remaining uncured resin was removed using ethanol. When both the magnetic particles and microcapsules were present, the structure became brittle. Therefore, a neodymium magnet was moved manually over the substrate to remove the uncured resin in the case of resin containing magnetic particles. Using this method, it was possible to remove the uncured resin efficiently while preventing damage to the structure. The curing depth and width of the resin were measured using a microscope (VHX-2000, Keyence, Osaka, Japan). The curing width was measured by focusing on the surface of the cover glass, whereas the curing depth was measured using a 3D reconstruction model while shifting the focus at regular intervals. Software provided with the microscope was used to build the 3D reconstruction model.

## Results

### Effects of magnetic particles on incident light

**Figure 2:**
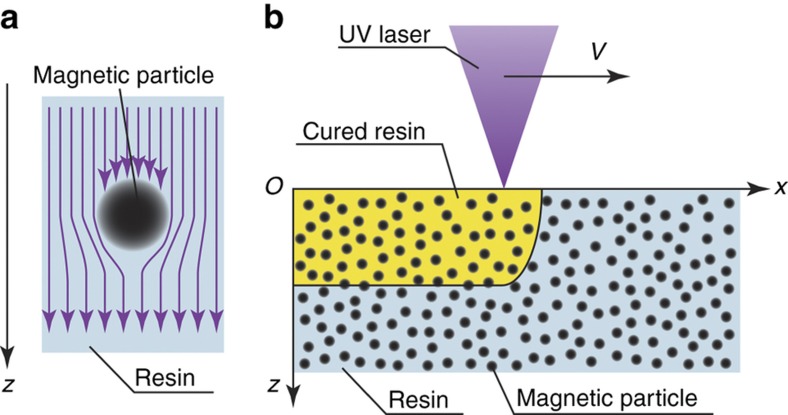
Curing model for a resin containing magnetic particles. (**a**) The effect of magnetic particles on the intensity of incident light. The purple arrows represent the incident light, whereas the black circles represent the magnetic particles. The incident light travels parallel to the *z*-axis. The magnetic particles absorb the incident light. In the proposed model, we assume that the extent of absorption is proportional to the cross-section of the magnetic particles. (**b**) Schematic of the laser-scanning process. The ultraviolet (UV) laser scans the surface of the resin containing magnetic particles at a velocity, *V*, along the *x* axis and solidifies the resin. The yellow area represents the solidified resin.

We modeled the photocurable resin containing magnetic particles. To begin, we assumed the situation shown in Figure 2a. In this case, uniform UV light, whose intensity is *I*^inc^(*x*, *y*, *z*, *t*), is irradiated on the resin containing magnetic particles. The depth of penetration of the UV light in the resin, which is the distance to which the light penetrates the resin, is *σ*_0_. The radius and density of the magnetic particles and the number of magnetic particles per unit volume are *a*, *ρ*, and *N*, respectively. The weight concentration of the particles, *w* (wt%), is used more widely than the number of particles per unit volume, *N*. Therefore, we used the following equation to convert *w* into *N*^9^:

(1)N(w)=w100−(1−ρ′ρ)wρ′ρ34πa3,

where *ρ′* is the density of the resin. The derivation of the above equation is given in the Supplementary Information.

Next, we considered the optical effects of the magnetic particles on the resin. Here we employed two approximations. First, we assumed that the magnetic particles absorb almost all the incident light. Second, we assumed that the shadow does not affect the absorption of other magnetic particles when it passes through them. We believe that this assumption is valid because the angle of the laser light varies corresponding to the numerical aperture, and it diffuses around the particle via diffraction. It is necessary to consider the influence of the shadow when the magnetic particle density or diameter is high.

On the basis of the abovementioned assumptions and Equation (1), we obtained the following equations for the light intensity:

(2)∂Iinc(x,y,z,t)∂z=−Iinc(x,y,z,t)σ,1σ=1σ0+Nπa2=1σ0+w100−(1−ρ′ρ)wρ′ρ34a.

Equation (2) shows that the transmitted light increases depending on the increase in the radius of the magnetic particles with a constant concentration *w*. Equation (2) makes it possible to determine the total penetration depth, *σ*, of light when the magnetic particles are present.

### Model of curing depth and width for resin containing magnetic particles

Next, we calculated the curing depth and width of the resin containing magnetic particles from Equation (2). In this case, the situation shown in Figure 2b was assumed. Here the UV laser beam focused by the lens travels along the *x* axis on the surface of the photocurable resin at speed *V* and cures the resin. The depth and width are labeled with the curing depth, *D*, and width, *W*, respectively. In this case, we assumed that the light intensity of the laser on the surface of the resin exhibited a Gaussian distribution. Under these conditions, the intensity of the incident light is given by

(3)Iinc(x,y,0,t)=I0exp(−(x−Vt)2+y2µ2),

where *I*_0_ and *μ* are the laser intensity at the center, and the radius of the laser beam, respectively. Using Equation (2), the intensity of the incident light in the resin can be calculated as

(4)Iinc(x,y,z,t)=I0exp(−(x−Vt)2+y2µ2−zσ)

To determine the curing area, we assumed that the resin becomes a solid if the exposure energy *E*(*x*, *y*, *z*) exceeds the threshold exposure energy, *E*_*T*_. The exposure energy *E*(*x*, *y*, *z*) is given by the time integral of the light intensity at position (*x*, *y*, *z*). On the basis of the existence of the threshold exposure energy, the area where

(5)E(x,y,z)≥ET

Change to slanting symbol is considered the solidified area. The integral of *I*^inc^(*x*, *y*, *z*, *t*) at position (*x*, *y*, *z*) becomes

(6)Einc(x,y,z)=∫−∞∞Iinc(x,y,z,t)dt=πI0µVexp(−y2µ2−zσ).

Given Equation (5), the curing depth, *D*, satisfies the relationship *E*^inc^(0, 0, *D*)=*E*_*T*_. Using this condition and Equations (1) and (6), one can determine the curing depth of the resin containing magnetic particles as follows:

(7)D=σ01+w100−(1−ρ′ρ)wρ′ρ3σ04alnπI0µETV.

Equation (7) indicates that the curing depth increases with an increase in the radius of the magnetic particles. Similarly, the curing width, *W*, satisfies the relationship *E*^inc^(0, *W*/2, 0)=*E*_*T*_, and we get

(8)W=2µlnπI0µETV.

Equation (8) shows that the curing width does not depend on the concentration of the magnetic particles.

### Measurement of penetration depth of resin to validate the proposed model

To verify whether the constructed model was accurate, the curing properties of the magnetic photocurable resin was measured. To use Equations (7) and (8) to determine the curing depth and width, respectively, it is essential to know the light penetration depth of the resin, *σ*_0_. Thus, we measured the *σ*_0_ value for a sample of the photocurable polymer SCR770 containing 5 wt% viscosity-increasing agent before validating the model. The viscosity-increasing agent was used to disperse the magnetic particles homogeneously^4^. We used Equation (7) to determine the penetration depth of the SCR770 sample containing the viscosity-increasing agent. In the absence of magnetic particles, the curing depth, *D*_0_, becomes

(9)D0=σ0lnπI0µETV.

Equation (9) indicates that if one were to measure the curing depth, *D*_0_, for different values of *I*_0_/*V* for the same *μ* and *E*_*T*_, *σ*_0_ can be calculated from the slope of the curve of *D*_0_ versus ln(*I*_0_/*V*).

**Figure 3:**
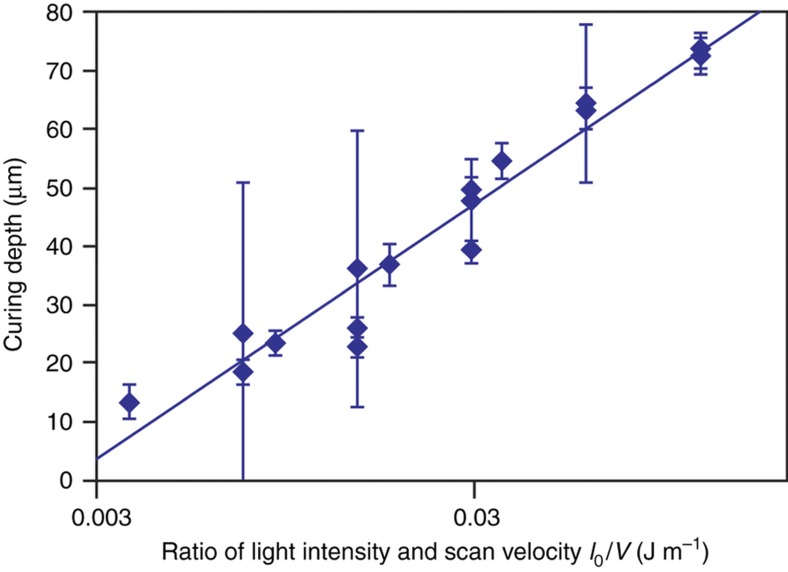
Measurement of the penetration depth of SCR770 *σ*_0_. The dots and error bars represent the average values and standard deviations of the curing depth, respectively. The light intensity ranged from 0.3 to 2.0 mW, and the scan speed ranged from 5 to 160 mm s^−1^. The average and standard deviation values were calculated from five measurements. The line represents the fitted curve, which suggested that the penetration depth of SCR770 was 19 μm, based on Equation (9). The number of measurements included four samples per point.

We measured the curing depth, *D*_0_, of the photocurable polymer SCR770 containing 5 wt% viscosity-increasing agent for laser intensities of 0.3–2.0 mW and scan speeds of 5–160 mm s^−1^. The procedure employed for measuring the curing width and depth is described in the MATERIALS AND METHODS section. Figure 3 shows the relationship between the measured *D*_0_ values and *I*_0_/*V*. With an increase in *I*_0_/*V*, the curing depth *D*_0_ increased. As expected based on Equation (9), *D*_0_ was proportional to ln(*I*_0_/*V*). From the slope of the curve in Figure 3, we calculated the penetration depth of SCR770 containing 5 wt% viscosity-increasing agent, *σ*_0_, and found it to be 19 μm.

### Measurement of curing depth and width of resin containing magnetic particles

Next, we measured the curing width and depth values of resin samples containing magnetic particles. Because Equation (7) shows that the curing characteristics depend on the diameter and density of the magnetic particles present in the resin, we used three types of magnetic particles, namely, FA-700 (ferrite), SeFeN (a rare metal), and magnetite particles. The characteristics of the magnetic particles used are listed in Table 1.

**Figure 4:**
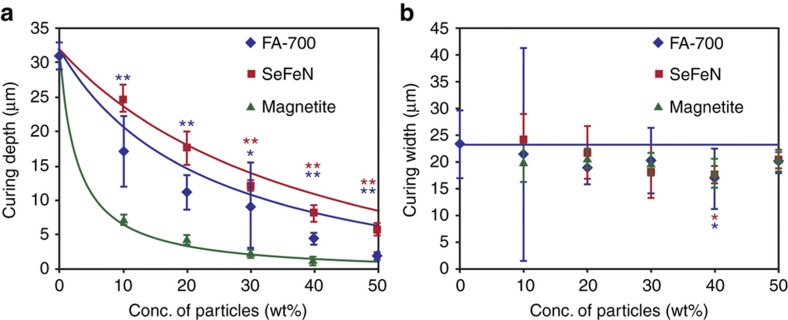
Curing characteristics for a resin containing magnetic particles. (**a**) Curing depths and (**b**) curing widths of the resin samples containing magnetic particles (FA-700/SeFeN/magnetite). The error bar represents the standard deviation. Five measurements were made for each point. The laser power and scan speed were 1 mW and 10 mm s^−1^, respectively. The fitted curves in **a** and **b** were calculated using Equations (7) and (8), respectively. For these calculations, we used the parameters listed in Table 1 as the material characteristics. The values of *D*_0_ and *W*_0_ used in the calculations were 32 and 23 μm, respectively. A single asterisk indicates that the *P* value was <0.05 compared to that in our model. Two asterisks indicate that the *P* value was less than 0.01 compared to that in our model. The number of measurements was four samples per point.

We prepared resin samples that contained 5 wt% viscosity-increasing agency and magnetic particles in concentrations of 0–50 wt%. Next, we measured the curing depth and width of these samples under the same optical conditions. The dots in Figure 4 present the obtained results. As shown in Figure 4a, the curing depth decreased gradually as the concentration of the magnetic particles increased. In addition, the curing depth was the smallest with magnetite particles. However, as shown in Figure 4b, the curing width remained almost constant for the three types of magnetic particles. The curves in Figure 4 were determined using Equations (7) and (8). The theoretical values were close to the experimental results, indicating that the proposed model is suitable for predicting the curing characteristics of resins containing magnetic particles.

There were a few inconsistencies between the results obtained from the model and the experiment. The theoretical value of the curing depth was higher than the experimental value. However, the curing width tended to slightly decrease with increasing concentration of the magnetic particles. We believe that this result is due to the brittle structure caused by the magnetic particles; consequently, the curing depth and width will both be low. To increase the accuracy of the theoretical prediction, it is necessary to consider the increased vulnerability of the material due to the magnetic particles.

The error bars for the samples with FA-700 at concentrations of 10 and 30 wt% in Figure 4a are large. This result is probably due to the differences in the degree of washing of the samples and not to the differences in their physical characteristics. It is likely that the variations in the measured values depend on how the uncured resin was removed, because the boundary between the hardened portion and the uncured portion is fragile. Washing away the uncured resin in the samples with FA-700 was perhaps not as skillful as in the case of samples with SeFeN and magnetite, as the FA-700 samples were first to be washed, and the washing technique improved with subsequent samples. As a result, the error bars for the samples with FA-700 are larger than those for the samples containing SeFeN or magnetite.

### Model for scattering of hollow microcapsules

Next, we modeled the effects of the hollow microcapsules on the curing characteristics. The microcapsules used consisted of a hydrocarbon covered with a polymer. Because the polymer and the hydrocarbon do not absorb a significant amount of light, the microcapsules scattered light via reflection and refraction. In addition, the diameter of the microcapsules was 30 μm, which is much larger than the wavelength of the laser used (325 nm). Thus, we can use geometric optics to calculate the extent of light reflection and refraction by the microcapsules. When the diameter of the particles is not significantly larger than the wavelength of the incident light, Mie theory should be used for calculating the extent of scattering^11^.

For the sake of simplicity, we assumed that the light scattering by the microcapsules was isotropic. If the refractive index of a medium after the incidence of light is larger than that before, the angle of the refracted light is larger than that of the incident light according to Snell’s law. Hence, the light that entered the microcapsules underwent reflection repeatedly and was emitted gradually in multiple directions. In the case where light scattering is anisotropic, the distribution of the scattered light should be calculated using ray tracing.

**Figure 5:**
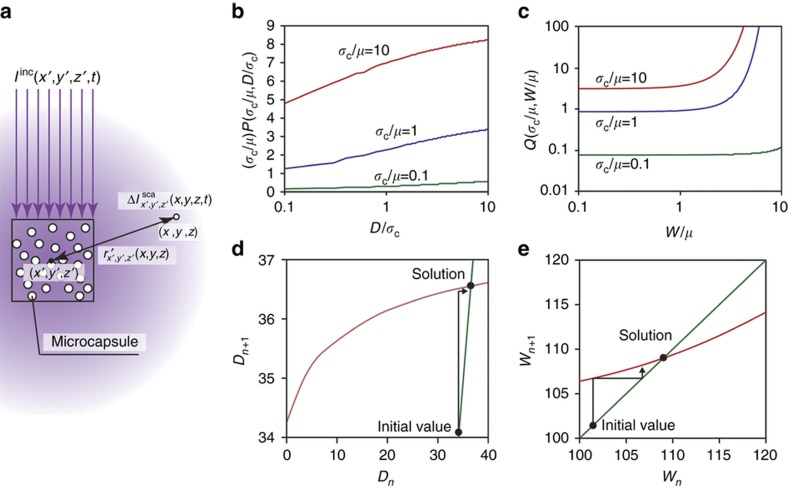
Calculation of curing depth and width for a resin containing microcapsules. (**a**) Schematic showing light scattering by the microcapsules. *I*^inc^(*x′*, *y′*, *z′*, *t*) and Δ*I*^*sca*^_*x′*,*y*′,__*z*′_(*x*, *y*, *z*, *t*) represent the incident light intensity at position (*x′*, *y′*, *z′*) and that of the light scattered at position (*x*, *y*, *z*) from position (*x′*, *y′*, *z′*), respectively. *r*’_*x*,*y*,*z*_(*x′*, *y′*, *z′*) is the distance between (*x′*, *y′*, *z′*) and (*x*, *y*, *z*). We assumed that the scattering of light by the microcapsules was isotropic. (**b** and **c**) Characteristics of the functions, *P* and *Q*, in Equations (23) and (24). (**a** and **b)** The *P* and *Q* functions, respectively. The integration ranges of *x* and *y* in function *P* were 0 to 10 for both variables. The upper end of the integration range of *z* in function *P* was 10. The integration ranges of *x*, *y*, and *z* in function *Q* were 0 to 10, −10 to 10, and 0 to 10, respectively. The integral width of *x*, *y*, and *z* in functions *P* and *Q* was 0.05. (**d** and **e**) The manner in which the curing width and depth were calculated, show how the sequences in Equations (25),Equations (26),Equations (27),Equations (28) converged. The thin green lines represent functions *D*_*n*+1_=*D*_*n*_ and *W*_*n*+1_=*W*_*n*_. The bold red lines represent the functions in Equations (25) and (27). The black lines represent the trajectories of the sequences in (25),(26)(27)(28).

As shown in Figure 5a, we assumed that the incident light *I*^inc^(*x′*, *y′*, *z′, t*) at position (*x′*, *y′*, *z′*) is scattered by the small volume, Δ*V′*, which contains microcapsules. Here the radius of the microcapsules and the number of microcapsules per unit volume are *a*_c_ and *N*_c_, respectively. We represent the intensity of the light scattered from position (*x′*, *y′*, *z′*) to position (*x*, *y*, *z*) as *ΔI*^sca^_*x′,y′,z*′_(*x*, *y*, *z*, *t*). Because we assumed that the light scattering by the microcapsules was isotropic, the intensity of the light scattered by the microcapsules in the small volume *ΔV’* becomes

(10)ΔIx′,y′,z′sca(x,y,z,t)=NcΔV′ac2Iinc(x′,y′,z′,t)4r'x,y,z2exp(−r′x,y,zσc),r′x,y,z=(x−x′)2+(y−y′)2+(z−z′)2,1σc=1σ+Ncπac2.

By integrating Equation (10) in space, the scattered light intensity at position (*x*, *y*, *z*) becomes

(11)I1inc(x,y,z,t)=Ncac24∫−∞∞∫−∞∞∫−∞∞Iinc(x′,y′,z′,t)r′x,y,z2exp(−r′x,y,zσc)dx′dy′dz′.

### Effect of multiple scattering

If the distance between the particles is shorter than the penetration depth of the resin, σ_c_, light scattering will occur several times, and the intensity of the light after the *i*-th scattering will be

(12)Iisca(x,y,z,t)=Ncac24∫−∞∞∫−∞∞∫−∞∞Ii−1sca(x′,y′,z′,t)r′x,y,z2exp(−r′x,y,zσc)dx′dy′dz′.

Here, the initial condition is

(13)I0sca(x,y,z,t)=Iinc(x,y,z,t).

From Equations (12) and (13), the light intensity after the *i*-th scattering can be computed. The sum of $I_{i}^{\mathrm{sca}}$becomes the total intensity of the scattered light *I*^sca^. Thus, we obtain

(14)Isca(x,y,z,t)=∑i=1∞Iisca(x,y,z,t).

Calculations that account for multiple scatterings involve infinite numbers of integration, which incurs a large computational cost. To reduce the computational cost, we assumed that the position where scattering occurred at the *i*-th position is the same as the position at which scattering occurred initially. In the case where scattering occurs far from the point of the first scattering, this approximation will not be valid. In that case, a Monte Carlo simulation^10^ or another approximation has to be used. Based on the above-mentioned assumption, Equation (12) takes the following simple form:

Iisca(x,y,z,t)=Ri−1Ncac24∫−∞∞∫−∞∞∫−∞∞Iinc(x′,y′,z′,t)r′x,y,z2(15)·exp(−r′x,y,zσc)dx′dy′dz′

where *R* is the ratio of the intensity of the rescattered light and is calculated as follows:

(16)R=Ncπac2σc.

The derivation method for the above equation is described in Supplementary Information. Spatial integral in Equation (15) does not depend on *i*. Thus, it is not necessary to spatially integrate each scattering, which helps reduce the computational cost. From Equations (1), (14), and (15), we obtain the total intensity of the scattered light as follows:

(17)Isca(x,y,z,t)=CNcac24∫−∞∞∫−∞∞∫−∞∞Iinc(x′,y′,z′,t)r′x,y,z2exp(−r′x,y,zσc)dx′dy′dz′,C=∑i=1∞Ri−1=11−Ncπac2σc=11−wc100−(1−ρ′ρc)wcρ′ρc3σc4ac,

where *w*_c_ and *ρ*_c_ are the concentration and density of the microcapsules, respectively. The parameter *C* in Equation (17) that represents multiple scattering shows that the effect of multiple scatterings becomes more pronounced with an increase in the penetration depth of the resin containing microcapsules *σ*_c_ and a decrease in the diameter of the microcapsules *a*_c_.

### Model for curing resin containing microcapsules using UV laser scanning

To calculate the curing depth and width of the resin containing microcapsules, we determined the exposure energy. Because the total light intensity is equal to the sum of the intensities of the incident and scattered light, we obtain the following equation:

(18)I(x,y,z,t)=Iinc(x,y,z,t)+Isca(x,y,z,t).

By integrating this with respect to time, we obtain

(19)E(x,y,z)=Einc(x,y,z)+CNcac24∫0∞∫−∞∞∫−∞∞Einc(x′,y′,z′)r′x,y,z2exp(−r′x,y,zσc)dx′dy′dz′,

where

(20)Einc(x,y,z)=πI0µVexp(−y2µ2−zσc).

Using Equations (5), (19), and (20), we can calculate the curing depth and width as follows:

(21)D=σclnπI0µETV(1+CNcac2σcP(σcµ,Dσc)),

(22)W=2µlnπI0µETV(1+CNcac2µQ(σcµ,Wµ)),

where

(23)P(σcµ,Dσc)=∫−D/σc∞∫0∞∫0∞1r2exp(−(σcµ)2y2−z−r)dxdydz,

(24)Q(σcµ,Wµ)=12∫0∞∫−∞∞∫0∞1r2exp(−y2−Wµy−z+rσc/µ)dxdydz.

The derivation of the detailed formulae is described in the Supplementary Information. The functions *P* and *Q* are integral functions whose values are always positive. The second term in Equation (22), which reflects the effect of scattering, indicates that the curing width increases as the effect of scattering becomes more pronounced. However, the curing depth is difficult to determine. The reason is as follows. Based on Equations (10) and (21), if the number of microcapsules per unit volume, *N*_c_, increases, the curing depth decreases because *σ*_c_ decreases. However, the second term in equation (21), which reflects the effect of scattering, will increase if *N*_c_ increases. The effect of light scattering on the curing depth is determined by both these effects.

To further understand the effect of light scattering on the curing depth and width, the calculated distributions of the dimensionless values (*σ*_c_/*μ*)*P* and *Q*, which reflect the degree of scattering in the depth and width directions, respectively, were plotted (see Figures 5b and c, respectively). As shown in Figure 5b, (*σ*_c_/*μ*)*P* increases with an increase in the curing depth *D*. This result occurs because the amount of light scattered to the deepest cured area increases in proportion to the curing depth. Furthermore, (*σ*_c_/*μ*)*P* increases with an increase in *σ*_c_, because the scattered light travels a large distance when *σ*_c_ is high. As is the case with the curing depth, the value of *Q* increases with an increase in the curing width, *W*.

### Calculation of curing depth and width of resin containing microcapsules

Because Equations (21) and (22) do not have analytical solutions, we need to solve them numerically. The solutions to Equations (21) and (22) are the convergence values of the following sequence:

(25)Dn+1=11+σ0Ncπac2+σ0Nπa2(D0+σ0ln(1+CNcac2σcP(σcµ,Dnσc))),

(26)D1=D01+σ0Ncπac2+σ0Nπa2,

(27)Wn+1=W01+4µ2W02ln(1+CNcac2µQ(σcµ,Wnµ)),

(28)W1=W0,

where *n* is a natural number. The reason this sequence converges is as follows. The colored lines in Figures 5d and e represent the left and right sides of Equations (25) and (27), respectively. The intersections of the lines in Figures 5d and e are the solutions to Equations (21) and (22), respectively. The black arrows in Figures 5d and e represent the procedures of the sequence in Equations (25)–(28). The sequences gradually approach the intersections, suggesting that the sequences that converge in Equations (25)–(28) are the solutions of Equations (21) and (22).

### Measurement of curing depth and width of photocurable polymer containing microcapsules

To validate our model for a resin containing hollow microcapsules, we investigated the effect of the microcapsules on the curing characteristics of a photocurable polymer. First, we prepared SCR770 samples containing a 5 wt% viscosity-increasing agent, 0–2 wt% microcapsules, and 0–30 wt% FA-700. Next, we measured the curing depths and widths of these samples under identical conditions.

**Figure 6:**
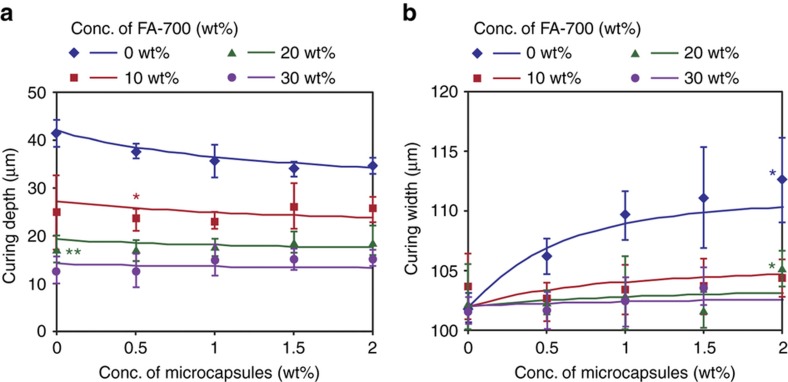
Curing characteristics for a resin containing microcapsules and magnetic particles. The (**a**) curing depth and (**b**) curing width. The laser power and scan speed were 2 mW and 10 mm s^−1^, respectively. The lines in **a** and **b** were determined using Equations (25)–(28). In these calculations, we used the parameters listed in Table 1. The values for the *D*_0_, *W*_0_ and μ parameters used were 41, 102, and 20 μm, respectively. A single asterisk indicates that the *P* value was <0.05 compared to that in our model. Two asterisks indicate that the *P* value was <0.01 compared to that in our model. The number of measurements was four samples per point.

Figure 6 shows the curing widths and depths of the samples containing different concentration of hollow microcapsules. As shown in Figure 6a, for the resin without any magnetic particles, the curing depth decreased as the concentration of the microcapsules increased. In the resin samples with an FA-700 concentration of >10 wt%, the curing depth increased slightly as the microcapsule concentration increased. The curing width also increased as the microcapsule concentration was increased, as shown in Figure 6b. The curing width of the resin samples with FA-700 contents of >10 wt% did not depend significantly on the concentration of the microcapsules. Inclusion of the magnetic particles prevented the scattered light of the microcapsules from traveling far. As a result, the increase in curing width by the microcapsules was suppressed in our model.

The lines in Figure 6 were determined using Equations (25)–(28). Visually, the theoretical and experimental values are largely in accordance. However, the experimental values for the curing width were higher than the theoretical values. We believe the cause of this discrepancy is the effect of the microcapsules. As the average radius of the microcapsules was 15 μm, there is a possibility that the curing width increased due to the existence of microcapsules protruding from the cured resin, which would have been included in the width measurements. Although there were a few differences between the theoretical and experimental results, as stated above, these differences were not large. Thus, the above-described effect could be ignored.

## Discussion

We elucidated the effects of magnetic particles and microcapsules on the curing characteristics of photocurable resins (Figures 4 and 6) and constructed a model for the curing of a resin containing magnetic particles and microcapsules (Figures 2 and 5). The model explains the obtained experimental results (Figures 4 and 6), indicating that it was an accurate model.

In the case of the model for the resin-containing magnetic particles, it was assumed that light was absorbed by the magnetic particles and that the shadow of the magnetic particles does not influence the light absorption by other magnetic particles. However, in the case of the model for the resin containing microcapsules, it was assumed that the light scattering was isotropic. If these assumptions do not match actual conditions, the proposed model will not yield satisfactory results. In such a case, Monte Carlo simulations based on Mie theory should be performed^10^. The advantage of the proposed model is that it can be expressed in a simple manner using Equations (7), (8), (21), and (22), and it supports an intuitive understanding of the phenomena involved, which is helpful when designing a resin that incorporates microparticles.

On the basis of this advantage, we considered optimizing the characteristics of the resins with magnetic particles and microcapsules. To discuss the effects of the magnetic particles on 3D fabrication, we defined the 3D fabrication efficiency for a resin as a high curing depth and low curing width result in a high aspect ratio during microstereolithography. Even at the same depth of curing, if the aspect ratio is higher, the curing width will be thinner. Thus, we defined the efficiency of fabrication as follows:

(29)η=Dσ04µ2W2.

Here, *η* is a dimensionless number and becomes 1 in the case where the resin does not contain any particles.

### Optimization of characteristics for resin containing magnetic particles

Next, we calculated the efficiency for a resin containing magnetic particles. Using Equations (1), (7), and (8), we could obtain the efficiency as follows:

(30)ηm=11+σ0πa2N=11+w100−(1−ρ′ρ)wρ′ρ3σ04a.

This expression indicates that the presence of magnetic particles lowers the fabrication efficiency. In addition, when the penetration depth, *σ*_0_, is low, it is possible to prevent a decrease in the efficiency due to magnetic particles. This result occurs because the resin is primarily responsible for light absorption. Furthermore, if the diameter of the particles present in the resin is larger, the efficiency will be higher.

For magnetically modifiable photocurable polymers, the number of magnetic particles per unit volume determines the magnetic properties^9^; this phenomenon can be expressed as follows:

(31)M=M0N43πa3

where *M* and *M*_0_ are the magnetizations of the resin with magnetic particles present and that of the magnetic particles themselves, respectively. Equation (31) shows that a decrease in the particle size has an adverse effect on the magnetic properties. To elucidate the effects of the fabrication efficiency and the magnetic properties, we calculated the efficiency using Equation (30) while assuming that *M* is equal to *M*_0_. This yielded

(32)ηm=4a4a+3σ0.

Equation (32) indicates that the diameter of the magnetic particles should be larger than *σ*_0_ for the fabrication efficiency to be high. In the case of large particles, however, attention should be paid to the lamination pitch. If the diameter of the particles is larger than the lamination pitch during microstereolithography, the particles will be removed by the squeegee. Based on these facts, the optimal size of the magnetic particles should be slightly smaller than the lamination pitch to ensure high-resolution fabrication and good material properties.

### Optimization of resin containing microcapsules

Next, we considered the case of a resin containing microcapsules. On the basis of Equations (21), (22), and (29), the efficiency in this case can be determined as follows:

(33)ηc=11+σ0Ncπac2D0+σ0ln(1+CNcac2σcP(σcµ,Dσc))D0+σ0ln(1+CNcac2µQ(σcµ,Wµ)).

Equation (1) and the first term, 1/(1+*σ*_0_*N*_c_*πa*_c_^2^), in Equation (33) indicates that the diameter of the microcapsules promotes fabrication efficiency, as does the diameter of the magnetic particles. Because an increase in *σ*_c_*P* increases *η*_c_, scattering in the depth direction does not adversely affect the fabrication efficiency. However, scattering in the width direction lowers the efficiency because *Q* exists in the denominator in Equation (33). A low *σ*_0_ negates the effect of light scattering because *σ*_c_ also decreases as *σ*_0_ decreases, and *σ*_c_*P* and *Q* decrease with a decrease in *σ*_c_ (Figures 5b and c). Furthermore, the first term, 1/(1+*σ*_0_*N*_c_*πa*_c_^2^), in Equation (33) also becomes small. Thus, when microcapsules and magnetic particles are used together, the fabrication efficiency improves. In fact, magnetic particles prevent the curing characteristics from being changed due to the scattering of light by the microcapsules (Figure 6).

Next, we discuss the light weight efficiency, including the effect of weight reduction by the microcapsules. The ratio of the volume occupied by the microcapsules per unit volume is as follows.

(34)ν=Nc43πac3

As shown from Equation (31), this form is the same as the magnetic particles. Replacing *N*_c_ in Equation (33) with Equation (34) yields the following equation.

(35)ηc=4ac4ac+3νσD0+σ0ln(1+C3ν4πacσcP(σcµ,Dσc))D0+σ0ln(1+C3ν4πacµQ(σcµ,Wµ)).

As shown in 4*a*_c_/(4*a*_c_+3*νσ*) in Equation (35), the efficiency increases as the diameter of the microcapsules increases. Furthermore, an increase in the diameter of the microcapsules serves to reduce the effect of scattering. Therefore, it is better for the microcapsule diameter to be as large as possible. Therefore, a diameter for the microcapsule that is slightly smaller than the laminating interval is appropriate.

### Optimization of resin containing typical microparticles

Finally, we discuss the development of resins containing typical microparticles. First, light absorption by the microparticles should be suppressed because it decreases the curing depth and the fabrication efficiency, as shown in Equation (30). Second, light scattering in the width direction should be suppressed. Light scattering in the width direction increases the curing width, and it also leads to a decrease in the efficiency. Finally, if the function of the polymer is proportional to the volume of the particles present, then the diameter of the particles should be larger than the penetration depth of the resin in accordance with Equations (32) and (35) to allow for high-resolution fabrication.

## Conclusion

In this report, we measured the curing characteristics of photocurable resins containing magnetic particles and microcapsules. We built a simple mathematical model to explain the experimental results. Using this mathematical model, the relationship between the particle properties contained in the photocurable resin and the photocurability is expressed via a mathematical formula. Using the simple formula, we showed how to optimize microparticles to obtain ideal characteristics, including photocurability, magnetic properties and density. To obtain ideal characteristics, it is preferable to make the particle size slightly smaller than the lamination interval and to suppress the scattering in the width direction. This model can be used for the development of a photocurable resin that incorporates microparticles.

## Figures and Tables

**Table 1:** Densities and diameters of FA-700, SeFeN, magnetite particles, microcapsules, and resin (SCR770)

Material	Density (g cm^−3^)	Diameter (μm)
FA-700	5.1	1.3
SeFeN	7.4	1.4
Magnetite	4.6	0.2
Microcapsule	0.03	30
Resin (SCR770)	1.17	—
