# Supplementary material for: Modeling and measurement of curing properties of photocurable polymer containing magnetic particles and microcapsules
Source: Microsyst Nanoeng. 2017 Aug 21;3:17035. doi: 10.1038/micronano.2017.35 (PMC6445025; doi:10.1038/micronano.2017.35)
Supplement: Supplementary Text [file micronano201735-s1.pdf]

Supplementary file

Modeling and measurement of curing properties of photocurable polymer containing magnetic particles and microcapsules

Yasui Masato[1] and Ikuta Koji[2]

Microsystems & Nanoengineering (2017) **3,** 17035; doi:10.1038/micronano.2017.35; Published online: 14 August 2017

RELATIONSHIP BETWEEN NUMBER OF PARTICLES PER UNIT VOLUME AND WEIGHT PERCENT

Let ρ' and ρ be the densities of the resin and micro particles, respectively. Let the particle radius be a. We assume that there is a mixed resin with concentrations of resin and micro particles of 100 —w (wt%) and w (wt%), respectively. If the weight of the mixed resins is 100, the number of particles n and the volume v in the mixed resin are given by

$$n = \frac{w}{\rho 4\pi a^3/3}, \tag{1S}$$

$$v = \frac{w}{\rho} + \frac{100 \quad w}{\rho'}. \tag{2S}$$

The number of particles per unit volume is given by dividing Equation (1S) by Equation (2S).

$$N(w) = \frac{n}{v} = \frac{w}{100 \quad \left(1 \quad \frac{\rho'}{\rho}\right)w}\frac{\rho'}{\rho}\frac{3}{4\pi a^3} \tag{3S}$$

CALCULATION OF RATIO OF INTENSITY OF RE-SCATTERED LIGHT

Let the light intensity I_0 illuminate the region of resin with the microcapsule. The radius and the density are a_c and N_c, respectively. At this time, the amount of light scattered by the capsule in the illuminated region is

$$E_0 = I_0 N_c \pi {a_c}^2. \tag{4S}$$

Because a spherical wave is assumed, the intensity of the scattered light is

$$I_{sca} = \frac{E_0}{4\pi r^2}\exp\left(\quad \frac{r}{\sigma_c}\right), \tag{5S}$$

where r and σ_c are the distance from the scattered region and the total penetration depth of the resin with microcapsules, respectively. Because the number of microcapsules between r and r+dr is $N_c 4\pi r^2 dr$, the amount of scattering light is $I_{sca}\pi a_c^2 N_c 4\pi r^2 dr$. By integrating in space, we can obtain the exposure energy as follows:

$$E_{sca} = \int_0^\infty I_{sca}\pi {a_c}^2 N_c 4\pi r^2 dr. \tag{6S}$$

By substituting Equation (5S) into Equation (6S), we can calculate as follows:

$$\begin{aligned} E_{sca} &= \textstyle\int_0^\infty \frac{E_0}{4\pi r^2}\exp\left(\quad \frac{r}{\sigma_c}\right)\pi {a_c}^2 N_c 4\pi r^2 dr \\ &= E_0\pi {a_c}^2 N_c \textstyle\int_0^\infty \exp\left(\quad \frac{r}{\sigma_c}\right)dr = E_0 \pi {a_c}^2 N_c \sigma_c. \end{aligned} \tag{7S}$$

The ratio of the intensity of the re-scattered light R is obtained by taking the ratio of Equation (7S) to Equation (4S).

$$R = \frac{E_{sca}}{E_0} = \pi {a_c}^2 N_c \sigma_c \tag{8S}$$

CALCULATION OF CURING DEPTH OF RESIN WITH MICROCAPSULES

Substituting Equation (19) into the conditional expression $E(0,0,D) = E_T$ yields the following

$$\begin{aligned} E_T = E^{inc}(0,0,D) + \frac{CN_c {a_c}^2}{4}\int_0^\infty \int_{\quad\infty}^\infty \int_{\quad\infty}^\infty \frac{E^{inc}(x', y', z')}{{r'_{0,0,D}}^2} \\ \cdot\exp\left(\quad \frac{r'_{0,0,D}}{\sigma_c}\right)dx'dy'dz'. \end{aligned} \tag{9S}$$

Further, substituting Equation (20) yields

$$\begin{aligned} E_T &= \tfrac{\sqrt{\pi}I_0\mu}{V}\exp\left(\quad \tfrac{D}{\sigma_c}\right) + \tfrac{CN_c {a_c}^2}{4}\textstyle\int_0^\infty \int_{\quad\infty}^\infty \int_{\quad\infty}^\infty \tfrac{1}{{r'_{0,0,D}}^2}\tfrac{\sqrt{\pi}I_0\mu}{V} \\ &\exp\left(\quad \frac{y'^2}{\mu^2} \quad \frac{z' + r'_{0,0,D}}{\sigma_c}\right)dx'dy'dz' \\ \Longleftrightarrow \frac{E_T V}{\sqrt{\pi}I_0\mu}\exp\left(\frac{D}{\sigma_c}\right) &= 1 + \frac{CN_c {a_c}^2}{4}\int_0^\infty \int_{\quad\infty}^\infty \int_{\quad\infty}^\infty \frac{1}{{r'_{0,0,D}}^2} \\ &\cdot\exp\left(\quad \frac{y'^2}{\mu^2} \quad \frac{z' \quad D + r'_{0,0,D}}{\sigma_c}\right)dx'dy'dz' \end{aligned} \tag{11S}$$

By converting the integral variables as $x' = \sigma_c x$, $y' = \sigma_c y$, $z' – D = \sigma_c z$, the following is obtained.

$$\begin{aligned} \tfrac{E_T V}{\sqrt{\pi}I_0\mu}\exp\left(\tfrac{D}{\sigma_c}\right) = 1 + \tfrac{CN_c {a_c}^2}{4}\textstyle\int_{\quad D/\sigma_c}^\infty \int_{\quad\infty}^\infty \int_{\quad\infty}^\infty \tfrac{\sigma_c}{r^2} \\ \cdot\exp\left(\quad \frac{{\sigma_c}^2}{\mu^2}y^2 \quad z \quad r\right)dx\,dy\,dz, r = \sqrt{x^2 + y^2 + z^2}. \end{aligned} \tag{12S}$$

Because the integral is symmetrical with respect to positive and negative values of x and y, it is possible to change the integral

[1]Laboratory for Cell Signaling Dynamics, RIKEN Quantitative Biology Center, 6-2-3 Furuedai, Suita, Osaka 565-0874, Japan and [2]Graduate School of Information Science and Technology, The University of Tokyo, 7-3-1 Hongo, Bunkyo-ku, Tokyo 113-8685, Japan
Correspondence: Ikuta Koji (ikuta@rcast.u-tokyo.ac.jp)

range from 0 to infinity.

$$\frac{E_T V}{\sqrt{\pi} I_0 \mu} \exp\left(\frac{D}{\sigma_c}\right) = 1 + C N_c a_c{}^2 \int_{D/\sigma_c}^{\infty} \int_0^{\infty} \int_0^{\infty} \frac{\sigma_c}{r^2} \cdot \exp\left(\quad \frac{\sigma_c{}^2}{\mu^2} y^2 \quad z \quad r \right) \mathrm{d}x\mathrm{d}y\mathrm{d}z \tag{13S}$$

Integration area is set by the P function in Equation (23), and it is summarized as follows:

$$\begin{aligned} &\frac{E_T V}{\sqrt{\pi} I_0 \mu} \exp\left(\frac{D}{\sigma_c}\right) = 1 + C N_c a_c{}^2 \sigma_c P\left(\frac{\sigma_c}{\mu}, \frac{D}{\sigma_c}\right) \\ &\Longleftrightarrow D = \sigma_c \ln \frac{\sqrt{\pi} I_0 \mu}{E_T V} \left(1 + C N_c a_c{}^2 \sigma_c P\left(\frac{\sigma_c}{\mu}, \frac{D}{\sigma_c}\right)\right). \end{aligned} \tag{14S}$$

CALCULATION OF CURING WIDTH OF RESIN WITH MICROCAPSULES

Substituting Equation (19S) into the conditional expression $E(0, W/2, 0) = E_T$ yields the following:

$$E_T = E^{\mathrm{inc}}(0, W/2, 0) + \frac{C N_c a_c{}^2}{4} \int_0^{\infty} \int_{\infty}^{\infty} \int_{\infty}^{\infty} \frac{E^{\mathrm{inc}}(x', y', z')}{r'_{0,W/2,0}{}^2} \cdot \exp\left(\quad \frac{r'_{0,W/2,0}}{\sigma_c} \right) \mathrm{d}x'\mathrm{d}y'\mathrm{d}z' \tag{15S}$$

Further, substituting Equation (20) yields

$$\frac{E_T V}{\sqrt{\pi} I_0 \mu} \exp\left(\frac{W^2}{4\mu^2}\right) = 1 + \frac{C N_c a_c{}^2}{4} \int_0^{\infty} \int_{\infty}^{\infty} \int_{\infty}^{\infty} \frac{1}{r'_{0,W/2,0}{}^2} \cdot \exp\left(\quad \frac{y'^2 \quad W^2/4}{\mu^2} \quad \frac{z' + r'_{0,W/2,0}}{\sigma_c} \right) \mathrm{d}x'\mathrm{d}y'\mathrm{d}z'. \tag{16S}$$

By converting the integral variables as $x' = \mu x$, $y' = \mu y + W/2$, $z' = \mu z$, the following is obtained.

$$\frac{E_T V}{\sqrt{\pi} I_0 \mu} \exp\left(\frac{W^2}{4\mu^2}\right) = 1 + \frac{C N_c a_c{}^2}{4} \int_0^{\infty} \int_{\infty}^{\infty} \int_{\infty}^{\infty} \frac{\mu}{r^2} \cdot \exp\left(\quad y^2 \quad \frac{W}{\mu} y \quad \frac{z + r}{\sigma_c/\mu} \right) \mathrm{d}x\mathrm{d}y\mathrm{d}z \tag{17S}$$

Because the integral is symmetrical with respect to positive and negative x, it is possible to change the integral range from 0 to infinity.

$$\frac{E_T V}{\sqrt{\pi} I_0 \mu} \exp\left(\frac{W^2}{4\mu^2}\right) = 1 + \frac{C N_c a_c{}^2 \mu}{2} \int_0^{\infty} \int_{\infty}^{\infty} \int_0^{\infty} \frac{1}{r^2} \cdot \exp\left(\quad y^2 \quad \frac{W}{\mu} y \quad \frac{z + r}{\sigma_c/\mu} \right) \mathrm{d}x\mathrm{d}y\mathrm{d}z \tag{18S}$$

The integration area is set by the Q function in Equation (24), and it is summarized as follows:

$$\begin{aligned} &\frac{E_T V}{\sqrt{\pi} I_0 \mu} \exp\left(\frac{W^2}{4\mu^2}\right) = 1 + C N_c a_c{}^2 \mu Q\left(\frac{\sigma_c}{\mu}, \frac{W}{\mu}\right) \\ &\Rightarrow W = 2\mu \sqrt{\ln \frac{\sqrt{\pi} I_0 \mu}{E_T V} \left(1 + C N_c a_c{}^2 \mu Q\left(\frac{\sigma_c}{\mu}, \frac{W}{\mu}\right)\right)}. \end{aligned} \tag{19S}$$
